# Circulating Plasmacytoid and Conventional Dendritic Cells Are Numerically and Functionally Deficient in Patients With Scrub Typhus

**DOI:** 10.3389/fimmu.2021.700755

**Published:** 2021-07-01

**Authors:** Seung-Ji Kang, Ki-Jeong Park, Hye-Mi Jin, Young-Nan Cho, Tae Hoon Oh, Seong Eun Kim, Uh Jin Kim, Kyung-Hwa Park, Sook-In Jung, Tae-Ok Kim, Hyo Shin Kim, Young-Goun Jo, Jae Kyun Ju, Seung-Jung Kee, Yong-Wook Park

**Affiliations:** ^1^ Department of Infectious Diseases, Chonnam National University Medical School and Hospital, Gwangju, South Korea; ^2^ Department of Rheumatology, Chonnam National University Medical School and Hospital, Gwangju, South Korea; ^3^ Department of Pulmonology, Chonnam National University Medical School and Hospital, Gwangju, South Korea; ^4^ Department of Surgery, Chonnam National University Medical School and Hospital, Gwangju, South Korea; ^5^ Department of Laboratory Medicine, Chonnam National University Medical School and Hospital, Gwangju, South Korea

**Keywords:** cytokine, Orientia tsutsugamushi, plasmacytoid dendritic cells, scrub typhus, conventional dendritic cells.

## Abstract

**Background:**

Dendritic cells (DCs) are specialized antigen-presenting cells known to bridge innate and adaptive immune reactions. However, the relationship between circulating DCs and *Orientia tsutsugamushi* infection is unclear. Therefore, this study aimed to examine the level and function of plasmacytoid DCs (pDCs) and conventional DCs (cDCs), two subsets of circulating DCs, in scrub typhus patients.

**Methods:**

The study included 35 scrub typhus patients and 35 healthy controls (HCs). pDC and cDC levels, CD86 and CD274 expression, and cytokine levels were measured using flow cytometry.

**Results:**

Circulating pDC and cDC levels were found to be significantly reduced in scrub typhus patients, which were correlated with disease severity. The patients displayed increased percentages of CD86^+^ pDCs, CD274^+^ pDCs, and CD274^+^ cDCs in the peripheral blood. The alterations in the levels and surface phenotypes of pDCs and cDCs were recovered in the remission state. In addition, the production of interferon (IFN)-α and tumor necrosis factor (TNF)-α by circulating pDCs, and interleukin (IL)-12 and TNF-α by circulating cDCs was reduced in scrub typhus patients. Interestingly, our *in vitro* experiments showed that the percentages of CD86^+^ pDCs, CD274^+^ pDCs, and CD274^+^ cDCs were increased in cultures treated with cytokines including IFN-γ, IL-12, and TNF-α.

**Conclusions:**

This study demonstrates that circulating pDCs and cDCs are numerically deficient and functionally impaired in scrub typhus patients. In addition, alterations in the expression levels of surface phenotypes of pDCs and cDCs could be affected by pro-inflammatory cytokines.

## Introduction

*Orientia tsutsugamushi* is an obligate intracellular bacterium that causes scrub typhus, a febrile illness widespread across the world (1). The disease initially exhibits typical eschar, rash, and if not managed sufficiently, fatal conditions including acute kidney injury, liver failure, meningoencephalitis, and multiple organ failure can develop (2, 3). Approximately a million patients are diagnosed each year in a broad area from the Asian-Pacific region, the so-called “Tsutsugamushi Triangle”, to Africa, Europe, and South America (1). Furthermore, it is spreading from rural to urban areas, which raises considerable concern in endemic countries (4).

Though the exact pathophysiology remains unclear, *O. tsutsugamushi* induces a range of dysregulated immune responses (5). The pathogen invades endothelial cells (ECs), monocytes, and dendritic cells (DCs), which are activated to secrete various cytokines and chemokines, provoking Th1 and Th2 dysregulation and the functional impairment of T lymphocytes (6–9). Recent investigations on unconventional immune cells such as mucosal-associated invariant T (MAIT) cells, natural killer (NK) cells, and natural killer T (NKT) cells suggest variations in frequency and function along with clinical relevance to the disease (10–12). Among these antigen-presenting cells (APCs), DCs are the most potent, central, and professional component that initiates and orchestrates immune reactions at the interface between innate and adaptive immunity (13). Currently, DCs can be classified into different subsets of conventional DCs (cDCs, formerly myeloid DC), plasmacytoid DCs (pDCs), monocyte-derived DCs (moDCs), and Langerhans cells based on their surface phenotype and functions (13–15). Of these different DCs, cDCs and pDCs are two main subsets of naturally occurring DCs that circulate in the peripheral blood. pDCs release type I interferon (IFN) against viruses, produce pro-inflammatory cytokines, and express major histocompatibility class (MHC) class II antigens and co-stimulatory molecules that activate numerous immune cells, including NK cells and NKT cells (16). cDCs are potent and specialized activators of T cells (13).

Several studies have described the relevance of DCs to scrub typhus. In one murine model, *O. tsutsugamushi* evaded autophagy and effectively invaded bone marrow-derived DCs, which showed impaired maturation and migration into lymphatic tissues (17). Another research study using human moDCs, a distinct DC population that matures during inflammation, reported that the pathogen replicated in moDCs, provoked the maturation of the cells, and triggered the secretion of cytokines, consequently stimulating CD4^+^ T cells (18). However, a study on the levels and functions of pDCs and cDCs in scrub typhus has yet to be conducted. Therefore, this study aimed to examine the levels and functions of pDCs and cDCs in scrub typhus, evaluate their clinical relevance, and investigate their roles under inflammatory conditions.

## Materials and Methods

### Study Subjects

The study cohort was comprised of 35 patients with scrub typhus (20 women and 15 men; mean age ± SD, 65.6 ± 15.4 years) and 35 healthy controls (HCs; 25 women and 10 men; mean age ± SD, 37.1 ± 7.5 years). The diagnosis of scrub typhus was performed by detecting *O. tsutsugamushi* antibodies in the patient’s serum using a passive hemagglutination assay kit (Genedia Tsutsu PHA II Test Kit; GreenCross SangA, Yongin, Korea). A positive result was defined as a titer of ≥ 1:80 in a single serum sample or at least a 4-fold rise in antibody titer at a follow-up examination, as described previously (10–12, 19). According to the number of dysfunctional organs, scrub typhus was graded into severe (≥ 2 organ dysfunctions), moderate (one organ dysfunction), and mild disease (no organ dysfunction) as previously described (20). The definition of organ dysfunction was: (1) renal dysfunction, creatinine ≥ 2.5 mg/dL; (2) hepatic dysfunction, total bilirubin ≥ 2.5 mg/dL; (3) pulmonary dysfunction, bilateral pulmonary infiltration on chest X-rays with moderate to severe hypoxia (PaO_2_/FiO_2_ < 300 mmHg or PaO_2_ < 60 mmHg or SpO_2_ < 90%); (4) cardiovascular dysfunction, systolic blood pressure < 80 mmHg despite fluid resuscitation; and (5) central nervous system dysfunction, significantly altered sensorium with a Glasgow Coma Scale (GCS) score of eight out of 15. All HCs were recruited in the same area (Jeollanam-do, South Korea) as the patients resided. HCs had no severe comorbidity such as malignancy, chronic liver, pulmonary, renal diseases, autoimmune disease, or fever within 72 hours prior to enrollment.

### Monoclonal Antibodies and Flow Cytometry

The following monoclonal antibodies (mAbs) and reagents were used in this study: fluorescein isothiocyanate (FITC)-conjugated Lineage Cocktail 1(CD3, CD14, CD16, CD19, CD20, CD56), phycoerythrin (PE)-conjugated anti-CD123, anti-CD86, and anti-CD274; allophycocyanin (APC)-conjugated anti-CD11c, anti-CD86, and anti-CD274; BV421-conjugated anti-HLA-DR; Alexa Fluor 647-conjugated anti-IFN-α; PE-conjugated anti-interleukin-12 (anti-IL-12), PE-Cy7-conjugated anti-tumor necrosis factor-α (anti-TNF-α) mAb, and PE-conjugated mouse IgG isotype control (all from Becton Dickinson, San Diego, CA, USA). The cells were stained with combinations of the appropriate mAbs for 20 minutes at 4°C. The stained cells were analyzed on a Navios flow cytometer using Kaluza software (version 1.5a; Beckman Coulter, Brea, CA, USA).

### Isolation of Peripheral Blood Mononuclear Cells (PBMCs) and Identification of pDCs and cDCs

Peripheral venous blood samples were collected in heparin-containing tubes, and PBMCs were isolated by density-gradient centrifugation using Ficoll-Paque Plus solution (Amersham Biosciences, Uppsala, Sweden). pDCs and cDCs were identified phenotypically as Lin1^-^HLA-DR^+^CD123^+^ cells and Lin1^-^HLA-DR^++^CD11c^+^ cells by flow cytometry, as previously described (21).

### Intracellular Cytokine Staining

Freshly isolated PBMCs (1 × 10^6^/well) were incubated in 1 mL of complete media, consisting of RPMI 1640, 2 mM _L_-glutamine, 100 units/mL of penicillin, and 100 μg/mL of streptomycin, and supplemented with 10% fetal bovine serum (FBS) for 2 hours in the presence of 10 μg/mL CpG (ODN2336; InvivoGen, San Diego, CA, USA) or 10 μg/mL non-CpG ODN control (InvivoGen) to stimulate pDCs and in the presence of 10 ng/mL IFN-γ (PeproTech, London, UK) and 2 μg/mL lipopolysaccharide (LPS; Sigma-Aldrich, St. Louis, MO, USA) to stimulate cDCs. For intracellular cytokine staining, 1 μL of brefeldin A (GolgiPlug; BD Biosciences, San Diego, CA, USA) was added for each 1 mL of cell culture. After incubation for an additional 4 hours, the cells were stained with FITC-conjugated Lineage Cocktail 1, PE-conjugated anti-CD123, APC-conjugated anti-CD11c, and BV421-conjugated anti-HLA-DR mAbs for 20 minutes at 4°C, fixed in 4% paraformaldehyde for 15 minutes at room temperature, and permeabilized with Perm/Wash solution (BD Biosciences) for 10 minutes. The cells were then stained with Alexa Fluor 647-conjugated anti-IFN-α, PE-conjugated anti-IL-12, and PE-Cy7-conjugated anti-TNF-α mAbs for 30 minutes at 4°C and analyzed by flow cytometry.

### Statistical Analysis

All comparisons of percentages, absolute numbers, cytokine levels, and expression levels of CD86 and CD274 in pDCs and cDCs were performed by analysis of covariance after adjusting for age and sex using the Bonferroni correction for multiple comparisons (ANCOVA). The Wilcoxon matched-pairs signed-rank test was used to compare changes in the cell numbers and expression of pDC and cDC phenotypes according to disease activity. To compare changes in surface phenotypes of pDCs and cDCs treated by pro-inflammatory cytokines, Kruskal-Wallis analysis with Dunn’s *post hoc* test was used for multiple comparisons. *P*-values of less than 0.05 were considered statistically significant. Statistical analysis was performed and graphs were generated using SPSS version 26.0 software (SPSS, Chicago, IL, USA) and GraphPad Prism version 5.03 software (GraphPad Software, San Diego, CA, USA), respectively.

## Results

### Subject Characteristics

The clinical and laboratory characteristics of 35 patients with scrub typhus are summarized in **Table 1**. According to disease severity based on the number of organs with dysfunction in these patients, 16 (45.7%), 12 (34.3%), and 7 (20.0%) had mild, moderate, and severe disease, respectively. Longitudinal monitoring was performed for 20 patients from the active state (before antibiotic therapy) to the remitted state (defined as resolution of all presenting symptoms after antibiotic therapy). Nine of those patients were available for measuring cell levels and surface phenotypes of circulating pDCs and cDCs.

**Table 1:** Clinical and laboratory characteristics of 35 patients with scrub typhus.

Variables	Scrub typhus	
Age, years, mean ± SD	65.6 ± 15.4	
Male/female, n	15/20	
Clinical variables, n (%)		
Fever	31 (88.6)	
Rash	24 (68.6)	
Eschar	28 (80.0)	
Confusion	5 (14.3)	
Severity of disease, n (%)		
Mild disease	16 (45.7)	
Moderate disease	12 (34.3)	
Severe disease	7 (20.0)	
Organ dysfunction, n (%)		
Renal dysfunction	2 (5.71)	
Hepatic dysfunction	4 (11.4)	
CNS dysfunction	4 (11.4)	
Respiratory dysfunction	14 (40.0)	
Circulatory dysfunction	7 (20.0)	
Comorbid conditions, n (%)		
Diabetes mellitus	8 (22.9)	
Cardiovascular disease	3 (8.57)	
Chronic kidney disease	1 (2.86)	
Chronic hepatic disease	1 (2.86)	
Chronic lung disease	0 (0.00)	
Malignancy	2 (5.71)	
Laboratory variables, mean ± SD		
Leukocyte count, cells/μL	7760 ± 3416	
Lymphocyte count, cells/μL	2109 ± 1169	
Hemoglobin level, g/dL	11.7 ± 1.7	
Neutrophil count, cells/μL	5008 ± 3198	
Platelet count, ×10^3^ cells/μL	173 ± 99	
Total bilirubin level, mg/dL	0.9 ± 0.6	
Total protein level, g/dL	6.0 ± 0.7	
Albumin level, g/dL	2.9 ± 0.6	
AST level, U/L	159 ± 178	
ALT level, U/L	122 ± 200	
Alkaline phosphatase level, U/L	192 ± 137	
LDH level, U/L	908 ± 276	
CRP level, mg/dL	10.4 ± 8.4	
Time at hospital visit*^a^*, days, mean ± SD	6.1 ± 3.3	

ALT, alanine aminotransferase; AST, aspartate aminotransferase; CNS, central nervous system; CRP, C-reactive protein; LDH, lactate dehydrogenase; n, number; SD, standard deviation.

^a^Time from symptom onset to antibiotic therapy.

### Reduced Numbers of pDCs and cDCs in Scrub Typhus Patients

**Figure 1:**
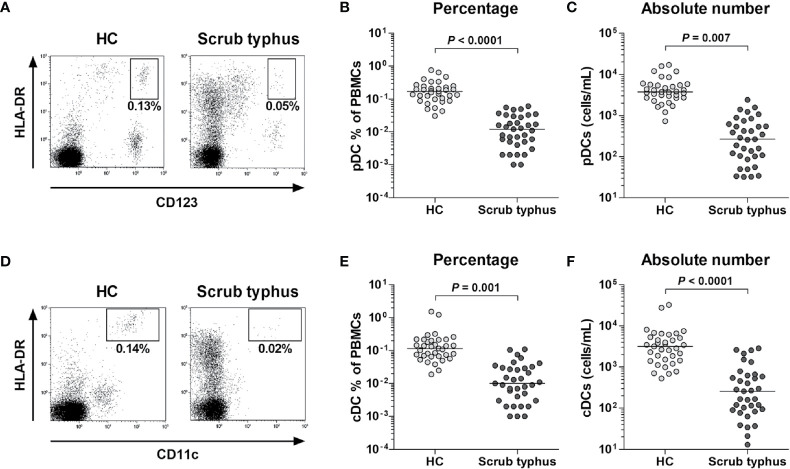
Decreased numbers of circulating pDCs and cDCs in peripheral blood samples of scrub typhus patients. Freshly isolated PBMCs from 35 HCs and 35 scrub typhus patients were stained with APC-conjugated anti-CD11c, BV421-conjugated anti-HLA-DR, FITC-conjugated Lineage Cocktail 1, and PE-conjugated anti-CD123 mAbs and then analyzed by flow cytometry. The percentages of pDCs and cDCs were calculated using a large gate including lymphocytes and monocytes. **(A, D)** Representative percentages of pDCs and cDCs determined by flow cytometry. **(B, E)** Percentages of pDCs and cDCs in PBMCs. **(C, F)** Absolute numbers of pDCs and cDCs (per milliliter of blood). The symbols represent individual subjects and the horizontal lines are median values. *P* values were calculated by ANCOVA test.

The percentage and absolute numbers of pDCs and cDCs in the peripheral blood samples of 35 scrub typhus patients and 35 HCs were determined by flow cytometry. pDCs and cDCs were defined as Lin1^-^HLA-DR^+^CD123^+^ cells and Lin1^-^HLA-DR^++^CD11c^+^ cells, respectively (**Figures 1A, D**). The percentage of circulating pDCs and cDCs were significantly lower in the patients than in the HCs (for pDCs, median 0.01% *versus* 0.17%, *P* < 0.0001; for cDCs, median 0.01% *versus* 0.12%, *P* = 0.001; **Figures 1B, E**). The absolute numbers of pDCs and cDCs were calculated by multiplying the pDC and cDC fractions in the mononuclear cells gate (using a flow cytometer) by absolute PBMC count (per milliliter of peripheral blood) determined using a standard hemocytometer. Scrub typhus patients had significantly lower absolute numbers of pDCs and cDCs than the HCs (for pDCs, median 267 cells/mL *versus* 3744 cells/mL, *P* = 0.007; for cDCs, median 259 cells/mL *versus* 3170 cells/mL, *P* < 0.0001; **Figures 1C, F**).

### Relationship Between pDC and cDC Levels and Clinical Parameters in Scrub Typhus Patients

To evaluate the clinical relevance of pDC and cDC levels in 35 patients with scrub typhus, we investigated the correlation between pDC and cDC percentages in the peripheral blood and clinical parameters by Spearman’s rank correlation analysis. The correlation results revealed that both circulating pDC and cDC percentages were significantly correlated with albumin levels (*P* = 0.003 and *P* = 0.019, respectively) and disease severity (*P* = 0.038 and *P* = 0.034, respectively). However, our experiments showed no significant correlation between circulating pDC and cDC percentages and leukocyte count, lymphocyte count, hemoglobin level, neutrophil count, platelet count, total bilirubin level, total protein level, aspartate aminotransferase level, alanine aminotransferase level, alkaline phosphatase level, lactate dehydrogenase level, or C-reactive protein level (**Table 2**).

**Table 2:** Spearman’s correlation coefficients for the percentages of pDCs and cDCs with respect to clinical and laboratory parameters in 35 patients with scrub typhus.

Variable	pDCs		cDCs	
	ρ	*P* value	ρ	*P* value
Age (years)	-0.215	0.215	-0.152	0.383
Leukocyte count (cells/μL)	0.030	0.863	-0.013	0.942
Lymphocyte count (cells/μL)	0.087	0.621	-0.045	0.796
Hemoglobin level (g/dL)	0.255	0.140	-0.038	0.827
Neutrophil count (cells/μL)	0.028	0.871	-0.044	0.802
Platelet count (×10^3^ cells/μL)	-0.106	0.545	-0.017	0.922
Total bilirubin level (mg/dL)	-0.136	0.459	-0.085	0.643
Total protein level (g/dL)	0.303	0.098	0.097	0.604
Albumin level (g/dL)	0.509	0.003^*^	0.411	0.019^*^
AST level (U/L)	-0.258	0.147	-0.087	0.632
ALT level (U/L)	0.089	0.623	0.280	0.114
Alkaline phosphatase level (U/L)	-0.146	0.418	-0.098	0.586
LDH level (U/L)	0.026	0.900	0.028	0.895
CRP level (mg/dL)	0.115	0.511	-0.171	0.326
Severity	-0.358	0.038^*^	-0.364	0.034^*^

ALT, alanine aminotransferase; AST, aspartate aminotransferase; CRP, C-reactive protein; LDH, lactate dehydrogenase; cDCs, conventional dendritic cells; pDCs, plasmacytoid dendritic cells; ρ, correlation coefficient.

^*^Indicates statistical significance.

### Activity of pDCs and cDCs in Scrub Typhus Patients

**Figure 2:**
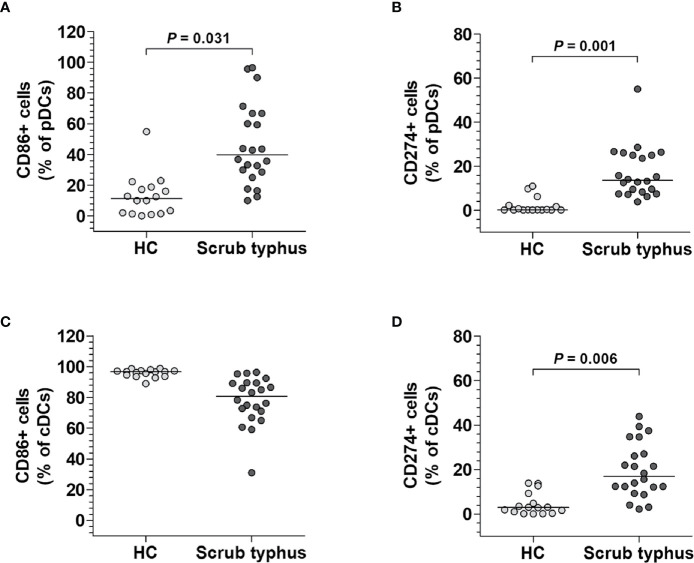
Phenotypes of circulating pDCs and cDCs in scrub typhus patients. Freshly isolated PBMCs were stained with APC-conjugated anti-CD11c, anti-CD86, and anti-CD274; BV421-conjugated anti-HLA-DR; FITC-conjugated Lineage Cocktail 1; and PE-conjugated anti-CD86, anti-CD123, and anti-CD274 mAbs and then analyzed by flow cytometry. **(A, C)**, Percentages of CD86-expressing pDCs and cDCs. **(B, D)**, Percentages of CD274-expressing pDCs and cDCs. The data were obtained from 16 HCs and 22 scrub typhus patients. The symbols represent individual subjects and the horizontal lines are median values. *P* values were calculated by ANCOVA test.

DCs can either induce or regulate immune reactions using specific molecules (14). Among those molecules, CD86 is known as a co-stimulatory marker that activates T cells, whereas CD274 is a co-inhibitory marker that restricts T cell function (14, 22). To examine the activity of both pDCs and cDCs, the expression levels of CD86 and CD274 molecules by each DC subset were compared between 22 scrub typhus patients and 16 HCs by flow cytometry. The percentages of both CD86^+^ and CD274^+^ pDCs were significantly higher in scrub typhus patients compared to the HCs (for CD86^+^ pDCs, median 39.9% *versus* 11.3%, *P* = 0.031; and for CD274^+^ pDCs, 13.6% *versus* 0.2%, *P* = 0.001; **Figures 2A, B**). The percentages of CD274^+^ cDCs were significantly higher in scrub typhus patients than in the HCs (median 17.0% *versus* 2.9%, *P* = 0.006; **Figure 2D**). However, no significant difference was observed in CD86^+^ cDC percentages between the patients and HCs (**Figure 2C**).

### Impaired Cytokine Production in pDCs and cDCs From Scrub Typhus Patients

**Figure 3:**
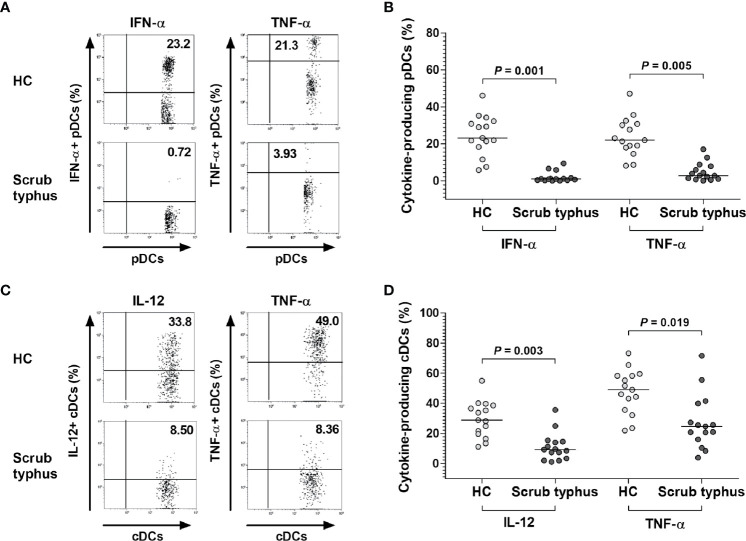
Decreased expression of IFN-α, IL-12, and TNF-α in pDCs and cDCs of scrub typhus patients. Freshly isolated PBMCs (1 × 10^6^/well) were incubated for 2 hours in the presence of CpG or the non-CpG ODN control for pDC stimulation, and in the presence of IFN-γ and LPS for cDC stimulation. **(A, C)** Representative IFN-α-, IL-12-, and TNF-α-expressing pDCs or cDCs as determined by flow cytometry. The data in **(B, D)** were obtained from 15 HCs and 15 patients with scrub typhus. The symbols represent individual subjects and the horizontal lines are median values. *P* values were calculated by ANCOVA test.

We next measured the levels of representative cytokines secreted by pDCs and cDCs from scrub typhus patients. The PBMCs from 15 scrub typhus patients and 15 HCs were incubated for 2 hours in the presence of CpG (for pDCs stimulation) or IFN-γ and LPS (for cDCs stimulation) and the expression levels of IFN-α, IL-12, and TNF-α in the pDC and cDC populations were examined at the single-cell level by intracellular flow cytometry (**Figures 3A, C**). The percentages of IFN-α^+^ and TNF-α^+^ pDCs were found to be significantly lower in scrub typhus patients than in HCs (for IFN-α^+^ cells, median 1.0% *versus* 23.2%, *P* = 0.001; for TNF-α^+^ cells, median 2.8% *versus* 22.0%, *P* = 0.005; **Figure 3B**). In addition, the percentages of IL-12^+^ and TNF-α^+^ cDCs were found to be significantly lower in scrub typhus patients compared to HCs (for IL-12^+^ cells, median 9.3% *versus* 28.8%, *P* = 0.003; for TNF-α^+^ cells, median 24.7% *versus* 49.0%, *P* = 0.019; **Figure 3D**).

### Changes in Levels and Surface Phenotypes of Circulating pDCs and cDCs According to Disease Activity

**Figure 4:**
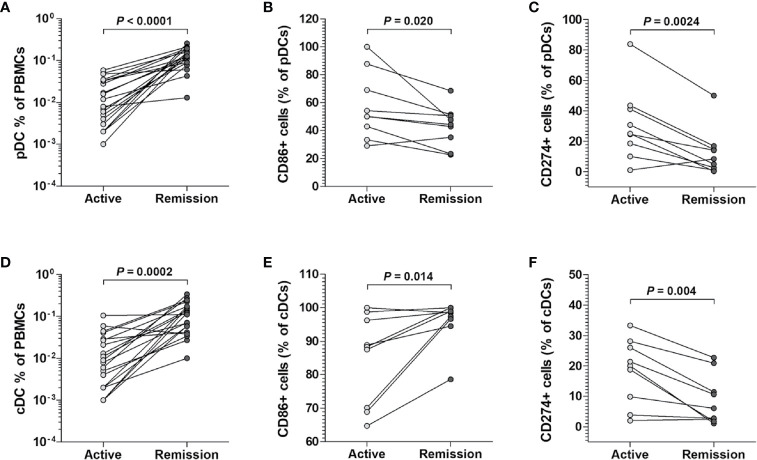
Changes in cell percentages and surface phenotypes of circulating pDCs and cDCs from scrub typhus patients. The percentages of pDCs **(A)** and cDCs **(D)** in the peripheral blood of 20 scrub typhus patients during active and remission states were determined by flow cytometry. The percentages of CD86-expressing **(B, E)**, and CD274-expressing **(C, F)** pDCs and cDCs were determined by flow cytometry. The data in **(B C, E, F)** were obtained from nine patients with scrub typhus. The symbols represent individual subjects. *P* values were calculated by Wilcoxon matched-pairs signed-rank test.

Based on our observation that the percentage of pDCs and cDCs was reduced during the active state of scrub typhus but the expression of CD86 and CD274 on pDCs and CD274 on cDCs was increased, we investigated whether changes in the proportion and surface phenotypes of pDCs and cDCs were related to disease activity. We found that the percentage of both pDCs and cDCs was greater when the disease was in remission than when it was active (for pDCs, median 0.13% *versus* 0.01%, *P* < 0.0001; for cDCs, median 0.12% *versus* 0.01%, *P* = 0.0002; **Figures 4A, D**). In the surface phenotypes, the percentages of CD86-expressing pDCs, and CD274-expressing pDCs and cDCs were lower in the remission state than in the active state (for CD86^+^ pDCs, median 44.3% *versus* 50.0%, *P* = 0.02; for CD274^+^ pDCs, median 8.4% *versus* 25.0%, *P* = 0.0024; for CD274^+^ cDCs, median 6.1% *versus* 20.0%, *P* = 0.004; **Figures 4B, C, F**). Conversely, the percentage of CD86-expressing cDCs was higher in the remission state compared to the active state (median 98.5% *versus* 88.4%, *P* = 0.014; **Figure 4E**).

### Effect of Stimulation With Pro-Inflammatory Cytokine Cocktail on the Activation of pDCs and cDCs

**Figure 5:**
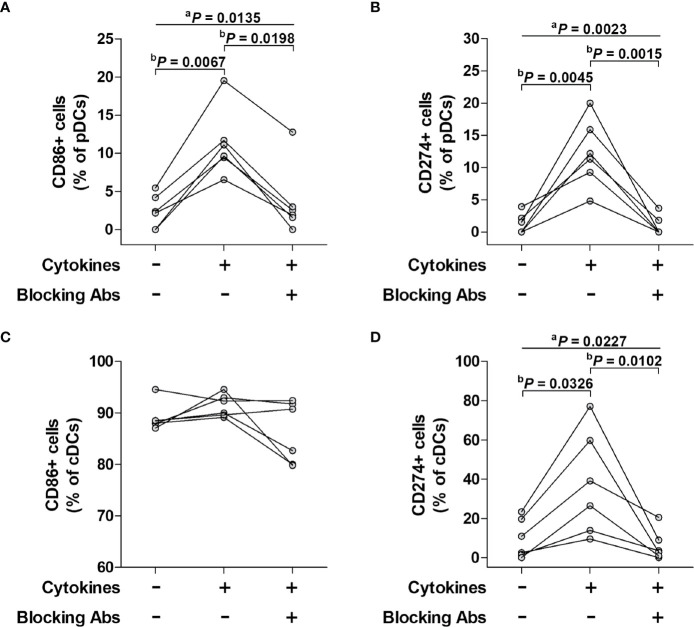
Effect of a pro-inflammatory cytokine cocktail and its blocking antibody on surface phenotypes of pDCs and cDCs. Freshly isolated PBMCs were incubated for 24 hours in the presence or absence of cytokine inhibitors (i.e., blocking antibodies against a cocktail of IFN-α, IL-12, and TNF-α and then stimulated with the cytokine cocktail for 16 hours. The stimulated cells were stained with APC-conjugated anti-CD11c, anti-CD86, and anti-CD274; BV421-conjugated anti-HLA-DR; FITC-conjugated Lineage Cocktail 1; PE-conjugated anti-CD86, anti-CD123, and anti-CD274 mAbs and then analyzed by flow cytometry. **(A, C)** Percentage of CD86-expressing pDCs and cDCs. **(B, D)** Percentage of CD274-expressing pDCs and cDCs. The data were obtained from six HCs. ^a^ *P* values among three groups were calculated by Kruskal-Wallis test. ^b^ *P* values between two groups were calculated by Dunn’s *post hoc* test.

To determine whether pDCs and cDCs could be activated by pro-inflammatory cytokines, PBMCs from six HCs were incubated for 24 hours in the presence or absence of cytokine inhibitors (i.e., blocking antibodies against a cocktail of IFN-γ, IL-12, and TNF-α) and then stimulated with a cytokine cocktail consisting of IFN-γ, IL-12, and TNF-α for 16 hours. Kruskal-Wallis analysis showed signficant differences among baseline, cytokine-treated, and blocking antibody-treated groups for the expression of CD86 (*P* = 0.0135) or CD274 (*P* = 0.0023) in pDCs and CD274 (*P* = 0.0227) in cDCs, except for the expression of CD86 (*P* = 0.1443) in cDCs. In addition, Dunn’s *post hoc* test was used for correction for multiple comparisons to analyze the differences between baseline and cytokine-treated groups or between cytokine-treated and blocking antibody-treated groups. The percentages of CD86^+^ pDCs were found to be significantly higher in cytokine-treated cultures compared to cytokine-untreated cultures (median 10.4% *versus* 2.3%, *P* = 0.0067), and then normalized to the untreated levels after treatment with blocking antibodies (median 10.4% *versus* 2.2, *P* = 0.0198; **Figure 5A**). The percentages of CD274^+^ pDCs significantly increased in cytokine-treated cultures compared to cytokine-untreated cultures (median 11.8% *versus* 0.8%, *P* = 0.0045), and then normalized to the untreated levels after treatment with blocking antibodies (median 11.8% *versus* 0.01%, *P* = 0.0015; **Figure 5B**). The percentages of CD274^+^ cDCs significantly increased in cytokine-treated cultures compared to cytokine-untreated cultures (median 32.8% *versus* 6.7%, *P* = 0.0326), and then normalized to the untreated levels after treatment with blocking antibodies (median 32.8% *versus* 3.2%, *P* = 0.0102; **Figure 5D**). However, the percentage of CD86^+^ cDCs remained unchanged regardless of cytokine stimulation or cytokine blocking (**Figure 5C**).

## Discussion

To the best of our knowledge, this was the first study to examine the levels and functions of pDCs and cDCs and assess the clinical relevance in scrub typhus patients. The patients exhibited decreased percentages and absolute numbers of circulating pDCs and cDCs, which was correlated with disease severity, and they had increased percentages of CD86^+^ pDCs, CD274^+^ pDCs, and CD274^+^ cDCs in the peripheral blood. These alterations in the levels and surface phenotypes of pDCs and cDCs were recovered in the remission state. In addition, the production of IFN-α and TNF-α by circulating pDCs, and IL-12 and TNF-α by circulating cDCs was reduced in scrub typhus patients. Interestingly, our *in vitro* experiments showed that the activation of pDCs and cDCs may be affected by pro-inflammatory cytokines.

In this study, scrub typhus patients displayed dramatic drops in circulating pDC and cDC levels, and this trend reflected disease severity. Consistent with the present study, septic shock patients displayed reductions in both pDCs and cDCs, which were associated with the development of intensive care unit infection or fatal outcomes (23, 24). A model that used *Citrobacter rodentium*-treated pDC-depleted mice showed increased systemic inflammation, and polymicrobial sepsis-induced cDC-depleted mice demonstrated increased mortality, indicating that pDCs and cDCs play protective roles in bacterial infections (25, 26). Collectively, these results suggest that the depletion of both pDCs and cDCs is a feature of infection-induced immune dysregulation, leading to the propagation and aggravation of systemic inflammation in scrub typhus infection.

Our data revealed enhanced co-stimulatory and co-inhibitory marker expression in both pDCs and cDCs. The expression of a co-stimulatory marker, indicated by CD86, was increased on pDCs during scrub typhus infection, which reflected their T-cell co-stimulatory function, consistent with previous studies on human immunodeficiency virus (HIV) and hepatitis C virus (HCV) infections (27, 28). *In vitro* experiments reported that *O. tsutsugamushi*-infected murine bone marrow-derived DCs or human moDCs exhibited increased CD86 expression (17, 18). Of note, the CD86^+^ cDCs were sustained at high levels regardless of the infection status, indicating that the T-cell priming function of cDCs was not solely dependent upon the scrub typhus infection. In contrast, the expression of a co-inhibitory marker, indicated by CD274, was higher in both pDCs and cDCs of scrub typhus patients than in those of HCs, in agreement with several studies on HIV and HCV infections (27, 28). Together, our findings suggest that both pDCs and cDCs expressed co-stimulatory and co-inhibitory molecules during scrub typhus infection, which could subsequently modulate the various immune reactions.

IFN-α, TNF-α, and IL-12 are well-known critical cytokines for controlling the intracellular growth of *O. tsutsugamushi* (5, 9, 29, 30). In the present study, the production of IFN-α and TNF-α by pDCs upon CpG stimulation was found to be diminished in scrub typhus patients. In addition, following IFN-γ/LPS stimulation, cDCs exhibited the reduced production of IL-12 and TNF-α in scrub typhus patients. Consistent with our study, the production of these cytokines by pDCs and cDCs was decreased after stimulation with TLR 7/8 ligands in HIV infection (27, 31). In contrast, Chu et al. reported increased IL-12 and TNF-α secretion from *O. tsutsugamushi-*infected human moDCs (18). Interestingly, the TNF-α expression in monocytes varied from study to study according to the strains and dose of *O. tsutsugamushi*, the nature of the infected cell line or tissue, and animal or human models. For example, low-dose *O. tsutsugamushi* infection was found to induce the inhibition of a pro-inflammatory pathway as well as the up-regulation of an anti-inflammatory pathway, promoting bacterial replication, whereas high-dose infection reversed this response, enhancing bacterial clearance (5). One possible explanation for the decreased pDC and cDC cytokine function in scrub typhus patients is the delayed or impaired maturation of pDC and cDC during *O. tsutsugamushi* infection, suppressing immune function. Another explanation is the hypo-responsiveness of preactivated human DCs to *in vitro* restimulation.

Our data revealed that the proportion of circulating pDCs and cDCs were significantly correlated with disease severity in scrub typhus patients. Consistent with our data, previous studies showed that only blood cDC levels but not pDC levels were inversely correlated with the severity of dengue virus infection and severe fever with thrombocytopenia syndrome (32, 33). Compared to viral infections, however, little is known about the correlation between disease severity and the numbers of circulating pDCs or cDCs in other bacterial infections. Our novel observation revealed that the numerical depletion of both pDCs and cDCs was recovered during the remission state of the infection, with changes in co-stimulatory and co-inhibitory surface marker expression. The recovery of pDC and cDC numbers strongly supports our data reflecting the inversed correlation between those cell numbers and disease severity. Moreover, the recovery of circulating pDC numbers was accompanied by a decreased expression of CD86 and CD274, suggesting that the antigen presentation or tolerogenic function of pDCs might be limited during the acute phase of the infection. This phenomenon was also firmly supported by our data demonstrating that pDC numbers were positively correlated with albumin levels, known as a negative acute phase reactant marker. Similar to pDCs, the expression of CD274 on cDCs was also decreased in the remission state, but the expression of CD86 was increased. Unlike pDCs, the difference in the CD86 expression pattern in cDCs might be due to the constitutionally high expression of CD86 in cDCs. Taken together, our findings indicate that DC activation and depletion reflects the severity of scrub typhus infection.

We hypothesized that a pro-inflammatory cytokine-rich environment could affect the expression of co-stimulatory and co-inhibitory markers in pDCs and cDCs during a scrub typhus infection. Our previous study reported the increased levels of cytokines such as IFN-γ, IL-12, and TNF-α in the early stage of a scrub typhus infection (11). After reproducing the pro-inflammatory milieu using a cytokine cocktail including IFN-γ, IL-12, and TNF-α, we observed the dynamics of CD86 and CD274 expression patterns in pDCs and cDCs, which was similar to our data in scrub typhus patients. These results suggest that the altered features of pDCs and cDCs might be secondary to the pro-inflammatory environment in the acute stage of the infection.

CpG-containing oligonucleotides (CpG ODNs) act on Toll-like receptor 9 (TLR9) that is expressed on B cells and pDCs to stimulate the innate immune system. Three types of stimulatory CpG ODNs have been identified: CpG-A, CpG-B, and CpG-C, which differ in their immune stimulatory activities. CpG-As are known to induce high production of type I IFN in pDCs but are not recognized in human B cells (34). Krug et al. have reported that CpG-A induced monocyte-derived DC-like phenotypes in PBMCs within 3 days but not such changes in purified monocytes (35). In the present study, however, CpG ODN 2336, a prototype of CpG-A, was used for culturing PBMCs for 2 hours, which was too short to induce significant changes in monocytes among PBMCs. Nonetheless, there are concerns that the cytokines that these cells produce may impact our DCs so in reality. It would have been optimal to sort the DCs prior to treatment.

There are some limitations in the present study. Based on recent advances through the emergence of powerful single-cell RNA sequencing and deep phenotyping technologies, originally three key DC subsets (cDC1, cDC2, and pDC) in peripheral blood have been expanded to six putative subsets (cDC1, cDC2-A, cDC2-B, CD16^+^ DC, Axl^+^ DC, and pDC) (36). In the present study, the antibody panels used for classification of circulating DCs comprised Lineage cocktail 1, CD123, HLA-DR, and CD11c, limiting our ability to distinguish among cDC subtypes or between pDCs and Axl^+^ DCs. CD11c positivity has been known as the marker selecting cDCs. Circulating Lin^-^HLA-DR^high^CD11c^+^ cells were defined as cDCs. By selecting CD11c, we may have excluded a subset of cDCs which are CD11c^low^ and are typically about 10% of total cDCs. This subset was mostly presumed to be cDC1, according to human blood and tissue dendritic cell phenotypes described by Rhodes et al. (36). Furthermore, CD16^+^ DC reported newly by Villani et al. also expressed CD11c (37). Some lineage marker panels included only CD3, CD19 and CD56, whereas ours additionally included CD14 and CD16. Thus, both classical (CD14^++^CD16^-^) and non-classical (CD14^+^CD16^++^) monocytes as well as T cells, B cells, and NK cells were negatively selected by our lineage panel. It is presumed that all monocyte subsets as well as CD16^+^ DCs might have been excluded from CD11c^+^ cDCs in the present study. Moreover, a number of computational flow cytometry tools such as FlowSOM, tSNE, oneSENSE, and ISOMAP have been developed to scale and represent high dimensional data of multiparametric flow cytometry (38). This approach could provide unbiased mapping and discovery of new cell phenotypes different from ones here identified using sequential manual gating. Further studies are warranted to answer this issue using unbiased gating strategy.

There are two methods for enumeration of absolute numbers of circulating DCs: dual-platform and single-platform. The dual-platform method is to calculate absolute numbers of circulating DCs by multiplying the percent amount of DCs in the mononuclear cells gate (using a flow cytometer) by absolute PBMC count determined using a standard hemocytometer. However, calculating absolute counts based on isolated PBMCs is not reliable, because PBMC isolations vary significantly from batch to batch. In contrast, the single-platform method, which is more accurate and easier than the former, is to enumerate absolute numbers of circulating DCs directly in a true count tube containing flow differential beads using a flow cytometer without hemocytometer. Unfortunately, the dual-platform method was used in the present study. The strategy used for quantification of absolute numbers is also to measure a fraction per volume (a relative metric).

In conclusion, this study firstly demonstrated that circulating pDCs and cDCs were numerically deficient and functionally impaired in scrub typhus patients. In addition, reduced cDC and pDC numbers reflected disease severity. We reported the novel finding that alterations in the expression levels of surface phenotypes of pDCs and cDCs could be affected by pro-inflammatory cytokines. These findings provide important insight into the dynamics of DC responses, which could present useful clues for future immunotherapy or vaccine development.

## Data Availability Statement

The original contributions presented in the study are included in the article/supplementary material. Further inquiries can be directed to the corresponding authors.

## Ethics Statement

The study protocol was approved by the Institutional Review Board of Chonnam National University Hospital. Written informed consent was obtained from all participants in accordance with the Declaration of Helsinki.

## Author Contributions

S-JiK, K-JP, H-MJ, Y-NC, TO, SK, UK, K-HP, S-IJ, T-OK, HK, Y-GJ, JJ, S-JuK, and Y-WP designed this study, collected clinical information, analyzed raw data, performed statistical analysis, and contributed to writing of the paper. S-JiK, K-JP, H-MJ, and Y-NC performed experiments. All authors contributed to the article and approved the submitted version.

## Funding

This work was supported by the National Research Foundation of Korea (2019R1A2C1003238, 2019R1I1A1A01040762), the Korea Health Technology R&D Project through the Korea Health Industry Development Institute funded by the Ministry of Health & Welfare, Republic of Korea (HI20C0079) and the Chonnam National University Hospital Biomedical Research Institute (CRI18042-21).

## Conflict of Interest

The authors declare that the research was conducted in the absence of any commercial or financial relationships that could be construed as a potential conflict of interest.
